# Thelen, Tatjana, Larissa Vetters and Keebet Benda‐Beckmann (eds.) 2017. Stategraphy: toward a relational anthropology of the state. Oxford: Berghahn Books. 163 pp. Pb.: £19.00. ISBN: 9781785337000.

**DOI:** 10.1111/1469-8676.12631

**Published:** 2019-06-06

**Authors:** Piyush Pushkar

**Affiliations:** ^1^ University of Manchester (UK)

Thelen, Vetters and Benda‐Beckmann attempt to do two things with this book. First, they offer us a new vantage point from which to investigate the state. Second, they offer case studies that showcase their theoretical focus. In this review, I start by explaining their argument. I then use some examples from the chapters in the book to demonstrate how their approach succeeds, and where they left me wanting more.

The editors argue that anthropology's ‘cultural turn’ led to a preponderance of work on representations of the state. Anthropologists following Gupta (1995) addressed this discursive construction of the state through the everyday practices of the people being studied. Thus a dichotomy was opened up between state images and practices.

To bridge this gap the editors suggest a focus on relations between social actors. They follow Gluckman in arguing that the stability of political systems has to be worked at, through the establishment and re‐establishment of ties between people. It is through a focus on these ties, on what happens between people, that state images, practices and formations can be brought into view.

The introduction succeeds in establishing their theoretical contribution and its usefulness for understanding the state. I have two quibbles. First, they mention Veena Das, without mentioning that her theoretical focus actually shares some similarities with theirs. That is, she investigates the state by focusing on how its representations emerge within concrete social relations. That said, the existence of another researcher with a similar mindset does not negate the value of their approach, even if it does take some shine off its novelty.

My second quibble is with the name they give to their theoretical approach: ‘stategraphy’. They do not explain the neologism. Since the suffix ‘graphy’ refers to writing, I understood stategraphy to mean something like ‘writing the state’, which perhaps would have been a more appropriate way to categorise the kind of anthropology that they are trying to move beyond, that is, an anthropology that focuses on representations at the expense of other foci of attention.

The case studies all focus on welfare services, and are all based in Europe, but spread over what they call ‘post‐socialist and post‐welfare states’ (p. 2). The editors and authors have clearly worked cooperatively with one another, as evidenced by interesting comparisons between chapters. For example, Dubois's chapter describes how French bureaucrats enact state welfare policies. Dubois's investigation of the uneasy relations between bureaucrats and citizens teases out how the bureaucrats’ moral judgements play a role in the citizens’ experiences of state policies. Forbess and James's chapter – based in the UK – then focuses on the ‘givers of advice’ who ‘have become increasingly essential to help people … interpret, mediate, or challenge the often inappropriate decisions made by the kinds of bureaucrats Dubois describes’ (p. 74). The comparison between different relations in different states thus helps in building a larger picture of how social actors understand and make states work.

Most chapters construct their arguments by layering intricately detailed ethnographic material with a wealth of contextual and historical information. This combination allows the authors to succeed in the editors’ stated aim of investigating how actors maintain images of state coherence by drawing on and reproducing old discourses, even as they transform them. Read thus gives fascinating new insights to how the boundaries between state and civil society are (re)produced in hospitals in the Czech Republic. Thelen, Thiemann and Roth do the same with state and kin in elder care in Serbia.

The pattern of investigating how actors put work into maintaining the stability of the state within the images they use in their relationships is replicated in all of the chapters. This replication, the shared focus of attention on welfare services, and the similarities of the arguments of the chapters contribute to the coherence of the volume. However, I wonder if the consistency of the contributors’ arguments has come at the expense of some ethnographic diversity. Perhaps the relational approach could also have revealed examples of social actors putting effort into maintaining an image of the state as dynamic or incoherent, rather than stable or coherent.

Finally, I must comment on the prose, which is at times dense, replete with floating nouns and pronouns that hinder intelligibility. This will make the introduction and some chapters somewhat inaccessible to undergraduate or lay readers. However, the persistent reader will be rewarded with a fresh theoretical approach to the state and, most importantly, richly detailed ethnographic case studies.
